# Genome-Wide Detection of Genetic Loci and Candidate Genes for Body Conformation Traits in Duroc × Landrace × Yorkshire Crossbred Pigs

**DOI:** 10.3389/fgene.2021.664343

**Published:** 2021-10-11

**Authors:** Hui Zhang, Zhanwei Zhuang, Ming Yang, Rongrong Ding, Jianping Quan, Shenping Zhou, Ting Gu, Zheng Xu, Enqin Zheng, Gengyuan Cai, Jie Yang, Zhenfang Wu

**Affiliations:** ^1^ College of Animal Science and National Engineering Research Center for Breeding Swine Industry, South China Agricultural University, Guangdong, China; ^2^ College of Animal Science and Technology, Zhongkai University of Agriculture and Engineering, Guangdong, China

**Keywords:** body conformation, carcass, crossbred pigs, GWAS, SNP

## Abstract

The Duroc × (Landrace × Yorkshire) hybrid pigs (DLY) are the most popular commercial pigs, providing consumers with the largest source of pork. In order to gain more insights into the genetic architecture of economically important traits in pigs, we performed a genome-wide association study (GWAS) using the GeneSeek Porcine 50 K SNP Chip to map the genetic markers and genes associated with body conformation traits (BCT) in 311 DLY pigs. The quantitative traits analyzed included body weight (BW), carcass length (CL), body length (BL), body height (BH), and body mass index (BMI). BMI was defined as BMI_CL_, BMI_BL_, and BMI_BH_, respectively, based on CL, BL, and BH phenotypic data. We identified 82 SNPs for the seven traits by GEMMA-based and FarmCPU-based GWASs. Both methods detected two quantitative trait loci (QTL) on SSC8 and SSC17 for body conformation traits. Several candidate genes (such as *TNFAIP3*, *KDM4C*, *HSPG2*, *BMP2*, *PLCB4*, and *GRM5*) were found to be associated with body weight and body conformation traits in pigs. Notably, the *BMP2* gene had pleiotropic effects on CL, BL, BH, BMI_CL_, and BMI_BL_ and is proposed as a strong candidate gene for body size due to its involvement in growth and bone development. Furthermore, gene set enrichment analysis indicated that most of the pathway terms are associated with regulation of cell growth, negative regulation of cell population proliferation, and chondrocyte differentiation. We anticipate that these results further advance our understanding of the genetic architecture of body conformation traits in the popular commercial DLY pigs and provide new insights into the genetic architecture of BMI in pigs.

## Introduction

In recent decades, pork has made up a large share of total worldwide meat production to accommodate growing human consumption. Growth and body conformation traits (such as body height and length) are economic traits which are moderately to highly important in pig production. It may be of interest to consider these traits in pig breeding schemes. Body height (BH) and body length (BL) are associated with meat production and were typical polygenic quantitative traits. Several studies have revealed significant single-nucleotide polymorphisms (SNPs) associated with BH or BL using the genome-wide association study (GWAS). For instance, Fan et al. (2009) (Fan et al., 2009) showed that *COL9A1*, *APOE*, *CART*, *INSL3*, and *DKFZ* were significantly associated with BL. Soma et al. (2011) (Soma et al., 2011) identified that four QTLs respectively on SSC4, SSC8, SSC13, and SSC14 were significantly associated with BL. Zhou et al. (2016) showed that *ss131324074* on SSC7 and *ss107849935* on SSC9 were significantly associated with BH, and *ss131389597* on SSC9 and *ss478942250* on SSC10 were significantly associated with BL in a Chinese Laiwu pig population (Zhou et al., 2016). An SNP (EU169095: g.40395T > G) within the *PPARδ* gene was found to be associated with the carcass length in a Large White × Meishan resource pig population (Xu et al., 2013). In addition, the number of thoracolumbar vertebrae can affect carcass length, which is an economically important trait in pig production. Rohrer and Keele (1998) (Rohrer and Keele, 1998) and Wada et al. (2000) (Wada et al., 2000) reported QTL for carcass length and vertebra number on the corresponding region of SSC 1. Mikawa et al. (2011) (Mikawa et al., 2011) proved that *VRTN* is the suspected cause of the heterogeneity of the number of vertebrae in commercial-breed pigs. Although previous findings have provided a certain number of molecular markers to help elucidate the genetic basis of swine body conformation traits, inadequacies and challenges remain when elucidating the biological mechanisms of the complex traits.

With the aid of high-density SNPs across the porcine genome, genome-wide association studies (GWASs) were utilized to dissect quantitative trait loci (QTLs) and genes associated with body conformation traits in pigs. The GWAS based on the mixed linear model (MLM) is the most popular method by taking account of population structure and genetic relatedness in deciphering the genetic architecture of complex traits in livestock (Sanchez et al., 2014). Multiple algorithms have been developed to boost both the computational efficiency and the statistical power of MLM methods (Kang et al., 2008; Zhou and Stephens, 2012). A recently developed GWAS model, named fixed and random model Circulating Probability Unification (FarmCPU) (Liu et al., 2016), has been widely used for detecting QTLs for economically important traits (Zhou J. et al., 2019; Tang et al., 2019; Wang et al., 2019). FarmCPU splits the MLM into separated fixed-effect and random-effect models and iteratively uses the two models to remove confounding, prevents model overfitting, and controls false positives simultaneously for an efficient computation (Liu et al., 2016). The major feature of FarmCPU is to correct for the effects of other markers by incorporating multiple markers simultaneously as covariates.

The Duroc × (Landrace × Yorkshire) hybrid pigs (DLY) are the most popular commercial pigs used in the Chinese pig industry. In this study, we performed GEMMA-based and FarmCPU-based GWASs for the body conformation traits including carcass length (CL), BL, and BH in 311 DLY pigs to identify the significant SNPs and then compare the SNP set respectively detected by the two methods. In addition, pigs provide a good animal model for studying the genetic basis of human disease due to their numerous physiological and phenotypic similarities with humans, including obesity. In human, the body mass index (BMI) is often used as an indicator to assess obesity (Locke et al., 2015), which is defined as a ratio of weight and height^2^ (Keys et al., 2014; Zhou et al., 2016). However, rare research has reported BMI differences in pigs (Zhou et al., 2016). Thus, we further analyzed the BMI in pigs and anticipated that this work will contribute to a better understanding of the genetic control of body conformation traits in agricultural animals and provide insights into the studies of human obesity and obesity-related diseases.

## Materials and Methods

### Experimental Animals and Phenotyping

Three-way crossbred DLY pigs intercrossed by Duroc boars and (Landrace × Yorkshire) sows were used in this study to conduct genetic analyses for body conformation traits. The experimental animals used in this study consisted of 311 DLY boars born in 2017 and were castrated at day 90 and raised in the same farm of Wen’s Foodstuffs Group Co., Ltd. (Guangdong, China). All pigs were fed with the same diets, raised under the same management conditions, and then slaughtered at 210 ± 3 days of age in a commercial abattoir in Shenzhen, Guangdong province. Three hundred eleven pigs were in the same batch. All the pigs were measured on the following traits: body weight (BW), body height (BH, from shoulder to ground), and body length (BL, from the midpoint of the ears to the tail head measured by a cloth tape). All the pigs were measured for BH and BL on the same flat ground before slaughter (Ma et al., 2009).

After skinning, scalding, scrapping, and eviscerating, carcass weight and carcass length (CL; measured from the first cervical vertebra to pubis) were measured within 30 min postmortem by the same person to minimize measurement errors. The ratio of weight and height^2^ shows relative independence from height or body length and high correlation with weight and fatness or subcutaneous fat (Keys et al., 2014; Zhou et al., 2016), as well as the abovementioned other weight/height ratios, such as weight/height, weight/height^3^, and height/weight^1/3^. The definition of human height seems to be different from the definition of pig height. In pigs, the measure of height should include the body length and hind leg length. Due to the differences in body structure between humans and pigs, a simple summation of body length and hind leg length seems inappropriate to be the “height” for BMI analysis in pigs. As described in previous studies, body length (Zhou et al., 2016) and body height (Gong et al., 2019) of pigs were treated as “height” to calculate the BMI (Supplementary Figure S1). In this study, in addition to BL and BH, we also added CL as the “height” of DLY to get the BMI value (respectively marked as BMI_CL_, BMI_BL_, and BMI_BH_) for subsequent analyses for a comprehensive understanding of the similarities and differences of different “height.”

### Genotyping and Quality Control

Genomic DNA was extracted from ear tissues using an animal tissue DNA extraction kit (Generay Biotech Co., Ltd., Shanghai, China) following the manufacturer’s protocol. DNA quality was detected using a NanoDrop ND-1000 (Peqlab Biotechnology) and agarose gel electrophoresis. The DNA concentration of the samples was adjusted to 50 ng/μl. Samples were genotyped with the GeneSeek Porcine 50 K SNP Chip (Neogen, Lincoln, NE, United States) (Ding et al., 2018). Quality control (QC) was carried out using PLINK v1.07 (Purcell et al., 2007) software. SNPs with call rates lower than 95%, ambiguous locations, and minor allele frequencies less than 0.01 were discarded. SNPs that failed the Hardy–Weinberg equilibrium test (*p* < 10^–6^) and unmapped or located on the sex chromosomes were also removed.

### Population Structure and Linkage Disequilibrium

Principal component analysis (PCA) was performed using the SNP dataset to assess the potential population stratification prior to conducting the GWAS. PCA was performed with the Genome-wide Complex Trait Analysis (GCTA) software (Yang et al., 2011) and Genome Association and Prediction Integrated Tool (GAPIT) (Lipka et al., 2012), respectively. Moreover, PLINK v1.07 (Purcell et al., 2007) was used to calculate the average linkage disequilibrium (LD) decay distance across the genome of the DLY pig population.

### Association Analyses

#### GEMMA-Based GWAS

The seven traits were analyzed using the same linear mixed model fitted in genome-wide efficient mixed-model analysis (GEMMA) software (Zhou and Stephens, 2012), one trait at a time. The statistical linear mixed model is described as follows:

 y=Wα+xβ+u+ε (1)

where y is an n×1 vector of phenotypes in the DLY pig population; α is a vector of the corresponding parameters including the intercept, sex, body weight (not included for BW), and the top five eigenvectors obtained prior to this analysis using the GCTA software (Yang et al., 2011); ***W*** is the incidence matrix of the appropriate dimension for the fixed effects; β is the effect of the marker; x is an n×1 vector of marker genotypes; u ∼ MVN (**0**, A $\sigma_{a}^{2}$ ) is an n×1 vector of animal residual additive genetic effect without accounting for the fitted SNP effects with ***A*** being the genomic relationship matrix estimated; and **ε** ∼ MVN (0, I $\sigma_{e}^{2}$ ) is the vector of residual errors, where $I_{n}$ is an n × n identity matrix.

#### FarmCPU-Based GWAS

The GAPIT (version 3.0) R package (Lipka et al., 2012) was used to conduct FarmCPU-based GWAS. All parameters were set as default. Briefly, the FarmCPU model consists of two parts: the fixed-effect model (FEM) and the random-effect model (REM), which is evaluated iteratively. The effects in the FEM include the top five principal components, sex, and pseudo quantitative trait nucleotides as (Liu et al., 2016; Tang et al., 2019), as follows:

$$y=Pb_{p}+M_{t}b_{t}+s_{j}d_{j}+e$$ (2)

where **y** is a vector of phenotypes of the analyzed trait; $b_{p}$ is a vector of fixed effects including top five principal components calculated by GAPIT, sex, and body weight (not included for BW); $b_{t}$ is a vector of the fixed effects for the pseudo QTNs (quantitative trait nucleotides); **P** and $M_{t}$ are the corresponding incidence matrices for $b_{p}$ and $b_{t}$ , respectively; $d_{j}$ is the effect of the **j-th** candidate SNP; $s_{j}$ is the genotype for the **j-th** candidate SNP; and ***e*** is a vector of the residuals.

The REM model updates the pseudo QTNs using the SUPER algorithm as follows (Wang et al., 2014):

y=u+e

where **y** is a vector of phenotypes, $u∼\text{MVN}\left(0,2K\sigma_{u}^{2}\right)$ with $\sigma_{u}^{2}$ being the unknown genetic variance and **K** being the kinship matrix computed by the pseudo-QTNs, and **e** is a vector of the residuals.

#### Identification of Significant Single-Nucleotide Polymorphisms Associated With body Conformations

Significant SNPs were identified for each trait as those that surpassed the threshold with a false discovery rate (FDR) controlled at 0.01 (Benjamini and Hochberg, 1995; Wang et al., 2017). Briefly, the threshold *p*-value was defined as follows:

P=FDR ×n/m

where *n* represents the number of SNPs with *p* < 0.01 in the GEMMA-based GWAS results, ordered ascendingly by their effects, and *m* is the number of qualified SNPs. The phenotypic variance explained by each significant SNP was estimated by the GCTA software (Yang et al., 2011). The Haploview v4.2 software (Barrett et al., 2005) was used to evaluate the LD pattern in specific genomic regions and conducted haplotype block analysis. The quantile–quantile (Q-Q) plots were generated to assess the influence of potential population stratification on GWAS using the GenABEL package (Aulchenko et al., 2007) after the top five principal components were added in the GWAS model.

### Identification of Candidate Genes and Functional Enrichment Analysis

Candidate genes were retrieved within 0.5 Mb on either side of the significant SNPs for the seven traits from the *Ensembl genome database version 99* of the *Sus scrofa* genome (Sscrofa11.1, http://jan2020.archive.ensembl.org, as of Jun 30, 2021) using the “biomaRt” package. Gene set enrichment analyses were conducted with these genes in the Metascape database (Zhou Y. et al., 2019). The terms with *p* < 0.01 were highlighted to further explore pathways and biological processes in which the genes are involved.

## Results

### Single-Nucleotide Polymorphism Genotyping and Phenotypic Variation

After QC, 38,398 SNPs with genotypes on 311 DLY pigs were retained for subsequent analyses. The descriptive statistics of BW, CL, BH, BL, and BMI for the 311 pigs are listed in Table 1. The phenotypic correlation coefficient between BW and BL (r = 0.55) was higher than that between BW and BH (r = 0.36) or CL (r = 0.31). The index shows a high correlation between BL and BH or CL (r ≥ 0.53), but lower between BH and CL (r = 0.35) (Supplementary Table S1). The phenotypic correlation coefficient between any two of these three BMI traits was high (r ≥ 0.69) (Supplementary Table S2).

**TABLE 1:** Variation of body weight and body conformation traits in DLY pigs.

Traits	N	Unit	Mean	SD	Min	Max	C.V. (%)
BW	311	kg	130.25	11.43	99.00	160.00	8.78
CL	310	cm	106.98	3.91	95.00	120.00	3.65
BL	310	cm	121.89	3.67	114.00	136.00	3.01
BH	310	cm	66.21	1.55	63.00	72.00	2.34
BMI_CL_	310	kg/m^2^	114.06	11.08	80.36	167.31	9.71
BMI_BL_	310	kg/m^2^	87.65	6.50	70.40	106.94	7.42
BMI_BH_	310	kg/m^2^	297.16	24.78	231.68	378.70	8.34

SD, standard deviation; CV, coefficient of variation.

### Population Structure and Linkage Disequilibrium

As shown in Supplementary Figure S2A, a slight genetic differentiation among the DLY pigs was observed, and the first five principal components were retrieved from *gapit* to reduce the influence of population stratification on the GWAS (Supplementary Figure S2B). All filtered SNPs were used to determine LD decay. At *r* ^2^ = 0.1, the LD decay distance decreases to 700 kb in the DLY population (Supplementary Figure S2C).

### Summary of GWASs Results for Body Conformation Traits

In total, 82 SNPs surpassing the threshold with an FDR controlled at 0.01 were identified by the two GWAS methods (Tables 2, 3, 4). Among them, there were seven SNPs significantly associated with BW, 15 SNPs with CL, 26 SNPs with BL, 14 SNPs with BH, 17 SNPs with BMI_CL_, 22 SNPs with BMI_BL_, and 13 SNPs with BMI_BH_. There were 53 significant SNPs found by GEMMA-based GWAS, 76 significant SNPs by FarmCPU-based GWAS, and 15 significant SNPs identified by both methods. Moreover, genes within the 1-Mb region of these significant SNPs were functionally annotated (Supplementary Table S3). The QQ plots of GWAS results are shown in Supplementary Figure S3.

**TABLE 2:** Description of SNPs significantly associated with BW in DLY pigs.

Traits	SSC^a^	SNP ID	Position (bp)^b^	MAF	*p*-value (MLM)	*p*-value (FarmCPU)	*r* ^2^/%^c^	Distance (bp)	Nearest gene
BW	1	*ASGA0001774*	26,631,978	0.44		5.17E-05	7.33	142,156	*TNFAIP3*
	1	*ASGA0005703*	215,031,222	0.23	7.38E-05		3.87	40,112	*KDM4C*
	1	*WU*_*10.2_1_306708221*	272,782,419	0.29	3.40E-05		5.32	12,164	*CEL*
	3	*ALGA0021159*	115,678,132	0.38		1.04E-04	5.18	—	—
	6	*M1GA0008725*	80,122,519	0.11		7.24E-05	6.3	164,248	*HSPG2*
	6	*WU_10.2_6_135404715*	146,999,505	0.25		7.41E-05	6.55	10,180	*DNAJC6*
	10	*ASGA0091894*	15,367,300	0.38	4.61E-05	3.90E-05	8.5	5,579	*MAP1LC3C*

The italic values were genes nearest the significant SNPs.

a Sus scrofa chromosome.

b The positions of the associated SNPs on the Sus Scrofa Build 11.1 assembly.

c Proportion of total phenotypic variation explained by each SNP.

**TABLE 3:** Description of SNPs significantly associated with CL, BL, and BH in DLY pigs.

Traits	SSC^a^	SNP ID	Position (bp)^b^	MAF	*p* -value (MLM)	*p* -value (FarmCPU)	*r* ^2^/%^c^	Distance (bp)	Nearest gene
CL	3	MARC0004652	6,498,219	0.16		1.00E–05	4.09	Within	*FAM200A*
	4	WU_10.2_4_3046732	3,559,394	0.14		3.34E–05	2.96	494,158	*TRAPPC9*
	4	WU_10.2_4_136884741	125,301,443	0.42		1.07E–06	3.31	within	*TGFBR3*
	7	WU_10.2_7_49907567	43,478,519	0.28		1.71E–08	6.31	118,773	*MMUT*
	7	ASGA0034652	81,310,017	0.09		2.46E–07	3.51	101,683	*RYR3*
	8	WU_10.2_8_25141199	24,043,163	0.14	9.03E–05		5.55	––	*––*
	12	ALGA0122685	43,952,687	0.42		1.23E–05	3.48	33,053	*KSR1*
	13	ALGA0067792	8,080,882	0.43		4.95E–05	2.12	155,446	*ZNF385D*
	13	WU_10.2_13_134401849	124,927,734	0.11	6.76E–05		4.79	8,421	*RTP1*
	14	14_12,070,780	10,892,893	0.12		7.03E–07	4.54	94,443	*STMN4*
	17	WU_10.2_17_17479009	15,827,454	0.37	2.28E–05		6.39	66,239	*BMP2*
	17	WU_10.2_17_17981232	16,253,154	0.33	1.60E–07	2.94E–06	9.74	491,286	*HA O 1*
	17	WU_10.2_17_18300615	1,6401737	0.39	6.63E–05		6.72	342,703	*HA O 1*
	17	MARC0028591	16,634,316	0.21	5.04E–05		5.37	110,124	*HA O 1*
	17	DBMA0000205	18,319,097	0.24	6.39E–05		6.37	83,449	*PLCB4*
BL	1	WU_10.2_1_168,922259	152,527,914	0.29		1.16E–04	1.06	65,735	*SOCS6*
	2	M1GA0024370	41,570,652	0.36		1.73E–05	6.91	Within	*OTOG*
	5	H3GA0015868	17,192,568	0.42		1.65E–05	0.95	23,004	*SCN8A*
	5	ALGA0031952	50,860,053	0.36		4.43E–06	4.7	54,759	*ETNK1*
	7	SIRI0000046	29,878,705	0.12		5.18E–05	5.6	Within	*ITPR3*
	7	ALGA0042427	65,595,703	0.36		9.20E–05	2.97	209,797	*EGLN3*
	7	ASGA0034393	65,625,414	0.36		9.20E–05	2.96	180,086	*EGLN4*
	8	ALGA0124320	96,665,022	0.09		5.22E–06	4.04	Within	*JADE1*
	10	ASGA0045707	740,406	0.22		3.89E–05	0.93	Within	*UCHL5*
	10	ALGA0056836	7,925,254	0.33		1.07E–08	4.94	Within	*SPATA17*
	12	WU_10.2_12_57752831	55,009,055	0.41		7.76E–07	1.86	37,840	*MYH13*
	13	WU_10.2_13_138014916	1,28,619,134	0.35	5.26E–05	1.20E–08	7.85	238	*CCDC50*
	14	ASGA0060896	7,192,572	0.26		9.86E–05	2.08	19,369	*PEBP4*
	14	ASGA0062769	37,378,781	0.42		2.94E–06	0.21	204,041	*TBX3*
	14	ALGA0077889	57,422,121	0.18		4.10E–06	7.16	2,783	*MAP3K21*
	14	ALGA0080935	108,956,441	0.17	1.15E–04	1.04E–04	8.94	2,435	*C10orf62*
	15	ALGA0085736	63,541,092	0.23	3.19E–05	1.15E–05	7.51	35,848	*NR4A2*
	17	ALGA0123867	13,717,308	0.26	7.91E–08	6.96E–08	13.04	11,990	*PRNP*
	17	WU_10.2_17_14580447	13,779,206	0.36	3.30E–06		8.3	5,914	*RASSF2*
	17	WU_10.2_17_15712448	14,734,253	0.17	5.59E–07		12.13	26,347	*SHLD1*
	17	ASGA0075536	15,196,027	0.35	7.42E–05		3.32	58,274	*FERMT1*
	17	WU_10.2_17_17075196	15,689,085	0.36	1.05E–05		9.56	60,750	*BMP2*
	17	WU_10.2_17_17013787	15,710,331	0.35	4.87E–05		7.11	39,504	*BMP2*
	17	WU_10.2_17_17479009	15827454	0.37	9.70E–07		8.41	66,239	*BMP2*
	17	DBMA0000205	18,319,097	0.24	1.24E–05		8.44	83,449	*PLCB4*
	17	DRGA0016692	29,138,019	0.24	6.14E–05		5.97	30,351	*XRN2*
BH	2	WU_10.2_2_9315312	9,766,370	0.44		2.72E–05	4.74	35,642	*DAGLA*
	4	MARC0012235	107,879,743	0.24	4.30E–06		6.36	23,759	*WNT2B*
	6	WU_10.2_6_13750573	13,837,643	0.33		3.80E–05	6.42	46,402	*VAC14*
	7	SIRI0000046	29,878,705	0.12		4.91E–05	4.87	Within	*ITPR3*
	7	ALGA0044383	105,954,725	0.17		2.67E–06	4.64	—	—
	9	ALGA0112140	21,921,748	0.36	7.13E–05		6.78	4,933	*GRM5*
	9	H3GA0027617	59,226,678	0.46	2.31E–05		5.95	188,970	*OPCML*
	13	WU_10.2_13_27755688	25,296,621	0.23		6.25E–06	3.39	Within	*ULK4*
	13	ALGA0073322	185,848,189	0.10	3.61E–05		5.7	168,304	*NCAM2*
	17	ALGA0123867	13,717,308	0.26	2.71E–05		7.08	12,423	*PRND*
	17	WU_10.2_17_15792357	14,642,640	0.26	9.69E–06		8.49	41,447	*SHLD1*
	17	WU_10.2_17_15712448	14,734,253	0.17	3.90E–05		7.24	26,347	*SHLD1*
	17	WU_10.2_17_17479009	15827454	0.37	9.23E–08	3.71E–08	9.5	66,239	*BMP2*
	17	DRGA0016582	15,949,323	0.36	1.12E–04		5	188,108	*BMP2*

The italic values were genes nearest the significant SNPs.

a *Sus scrofa* chromosome.

b The positions of the associated SNPs on the *Sus scrofa* Build 11.1 assembly.

c Proportion of total phenotypic variation explained by each SNP.

**TABLE 4:** Description of SNPs significantly associated with BMI_CL_, BMI_BL_, and BMI_BH_ in DLY pigs.

Traits	SSC^a^	SNP ID	Position (bp)^b^	MAF	*p*-value (MLM)	*p*-value (FarmCPU)	*r* ^2^/%^c^	Distance (bp)	Nearest gene
BMI_CL_	4	WU_10.2_4_3046732	3,559,394	0.14		1.16E–05	3.1	494,158	*TRAPPC9*
	4	ASGA0017873	7,875,059	0.48	7.49E–05		6.17	within	*ST3GAL1*
	4	WU_10.2_4_136884741	125,301,443	0.41		6.59E–06	3.13	within	*TGFBR3*
	7	WU_10.2_7_49907567	43,478,519	0.27		1.02E–09	6.12	118,773	*MMUT*
	7	ASGA0034652	81,310,017	0.09		6.86E–07	3.14	101,683	*RYR3*
	12	ALGA0122685	43,952,687	0.42		4.67E–05	2.96	33,053	*KSR1*
	14	DIAS0004697	10,274,713	0.49	1.01E–04		5.94	within	*PPP2R2A*
	14	ASGA0061212	10,327,418	0.48	8.01E–05		5.89	within	*BNIP3L*
	14	14_12070780	10892893	0.12	9.54E–05	1.11E–05	4.72	94,443	*STMN4*
	14	ASGA0066313	116,729,015	0.19		6.97E–06	5.42	—	—
	15	WU_10.2_15_136106170	122,789,034	0.49		4.76E–05	1.57	—	—
	17	WU_10.2_17_16861730	15,492,508	0.42	7.23E–05		5.38	257,327	*BMP2*
	17	WU_10.2_17_17479009	15,827,454	0.37	3.76E–06		6.3	66,239	*BMP2*
	17	WU_10.2_17_17981232	16,253,154	0.33	1.42E–08	4.32E–07	10.13	491,286	*HA O 1*
	17	MARC0028591	16,634,316	0.21	1.07E–04		5.36	110,124	*HA O 1*
	17	ALGA0093478	16,919,581	0.40	1.50E–05		6	38,251	*TMX4*
	17	DBMA0000205	18,319,097	0.24	6.89E–06		7.75	83,449	*PLCB4*
BMI_BL_	2	M1GA0024370	41,570,652	0.36		1.26E–06	7.47	within	*OTOG*
	3	ALGA0020800	108,685,228	0.28		2.34E–07	5.34	5,650	*LCLAT1*
	3	WU_10.2_3_117349436	110,594,886	0.18		7.67E–07	2.46	11,035	*PLB1*
	4	ALGA0023916	20,318,986	0.35		9.07E–06	4.7	within	*SAMD12*
	5	H3GA0015868	17,192,568	0.42		4.25E–08	10.14	23,004	*SCN8A*
	5	ALGA0031952	50,860,053	0.36		6.70E–05	4.2	54,759	*ETNK1*
	7	SIRI0000046	29,878,705	0.12	3.25E–05	1.62E–08	5.9	within	*ITPR3*
	7	ASGA0034397	65,649,418	0.17		5.35E–05	0.14	156,082	*EGLN3*
	8	ALGA0124320	96,665,022	0.09		9.75E–06	4.23	within	*JADE1*
	10	ALGA0056836	7,925,254	0.33		1.60E–06	4.95	within	*SPATA17*
	10	ALGA0106806	41,215,023	0.41		2.92E–06	1.67	37,046	*SVIL*
	11	ALGA0061436	23,388,539	0.27		4.79E–05	1.08	within	*ENOX1*
	12	WU_10.2_12_57752831	55,009,055	0.41		3.12E–06	1.78	37,840	*MYH13*
	13	WU_10.2_13_138014916	1,28,619,134	0.35	4.39E–05	2.45E–09	7.5	238	*CCDC50*
	14	ALGA0080935	108,956,441	0.17		2.38E–05	8.83	2,435	*C10orf62*
	15	ALGA0085736	63,541,092	0.23		1.81E–05	7.81	35,848	*NR4A2*
	17	ALGA0123867	13,717,308	0.26	1.57E–06	1.01E–07	13.14	11,990	*PRNP*
	17	WU_10.2_17_14580447	13,779,206	0.36	5.82E–05		8.38	5,914	*RASSF2*
	17	WU_10.2_17_15712448	14,734,253	0.17	1.30E–06		12.06	26,347	*SHLD1*
	17	WU_10.2_17_17075196	15,689,085	0.36	9.11E–05		10.14	60,750	*BMP2*
	17	WU_10.2_17_17479009	15827454	0.37	1.36E–06		8.59	66,239	*BMP2*
	17	DBMA0000205	18,319,097	0.24	2.14E–05		8.81	83,449	*PLCB4*
BMI_BH_	2	MARC0035424	149,466,993	0.09		2.66E–05	4.22	717	*SPINK6*
	4	ASGA0022193	111,702,541	0.08	2.00E–06	5.34E–07	7.49	68,270	*SLC25A24*
	7	ASGA0031627	19,251,635	0.11		7.45E–05	4.15	within	*MRS2*
	8	MARC0065833	76712691	0.1	3.27E–05	4.92E–06	5.7	13,340	*FBXW7*
	8	WU_10.2_8_80223477	75,732,460	0.1	3.46E–05	5.69E–06	5.69	within	*MND1*
	8	H3GA0025014	76,555,090	0.11		1.04E–05	5.22	within	*FBXW7*
	8	ASGA0039051	74,641,551	0.1		1.32E–05	4.4	23,047	*DCHS2*
	8	MARC0109188	73,439,961	0.1		2.15E–05	4.29	62,703	*FRAS1*
	8	WU_10.2_8_80208219	75,717,201	0.11		2.72E–05	5	within	*MND1*
	8	ALGA0048253	74,373,748	0.09		5.30E–05	3.55	within	*RBM46*
	12	ASGA0054390	36,455,949	0.11		3.40E–05	4.38	within	*BRIP1*
	17	ALGA0092770	4,018,290	0.12		8.18E–05	3.32	78,564	*MSR1*
	18	WU_10.2_18_18,355,722	17,275,733	0.14	4.89E–05		5.58	226,122	*MKLN1*

The italic values were genes nearest the significant SNPs.

a *Sus scrofa* chromosome.

b The positions of the associated SNPs on the *Sus scrofa* Build 11.1 assembly.

c Proportion of total phenotypic variation explained by each SNP.

### Body Weight

**FIGURE 1:**
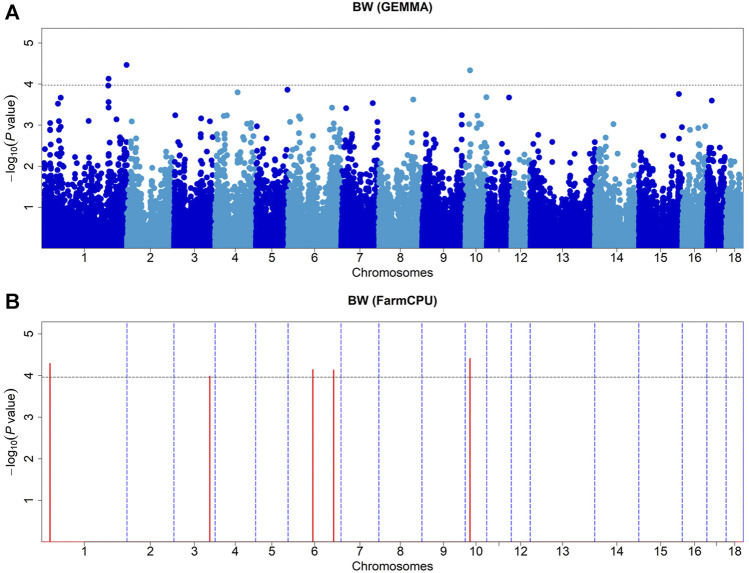
Manhattan plots of GEMMA-based GWAS **(A)** and FarmCPU-based GWAS **(B)** for BW in DLY pigs. The *x*-axis represents the chromosomes, and the *y*-axis represents the −log10 (*p*-value). The dashed lines indicate the thresholds for BW in DLY of GEMMA-based GWAS (*p* = 1.07E-04) and FarmCPU-based GWAS (*p* = 1.10E-04).

We identified seven SNPs that were significantly associated with body weight, located on chromosomes 1, 3, 6, and 10, respectively (Table 2). The GEMMA-based GWAS detected three of these SNPs, and the FarmCPU-based GWAS detected five of them (Figure 1A,B). Of them, four SNPs were located within the previously reported QTLs for BW (https://www.animalgenome.org/cgi-bin/QTLdb/SS/index/). *ASGA0091894* is the only SNP detected by both methods, which is significantly associated with BW. There were three SNPs, *ASGA0001774*, *ASGA0005703*, and *WU_10.2_6_135404715*, which showed significant effects on body weight, which otherwise was not reported previously. The most significant SNP detected by MLM and FarmCPU was *WU_10.2_1_306708221* (*p* = 3.40 × 10^−5^) on SSC1, and *ASGA0091894* (*p* = 3.90 × 10^−5^) explained 5.32 and 8.5% of the phenotypic variance of BW, respectively.

### Carcass Length, Body Length, and Body Height

**FIGURE 2:**
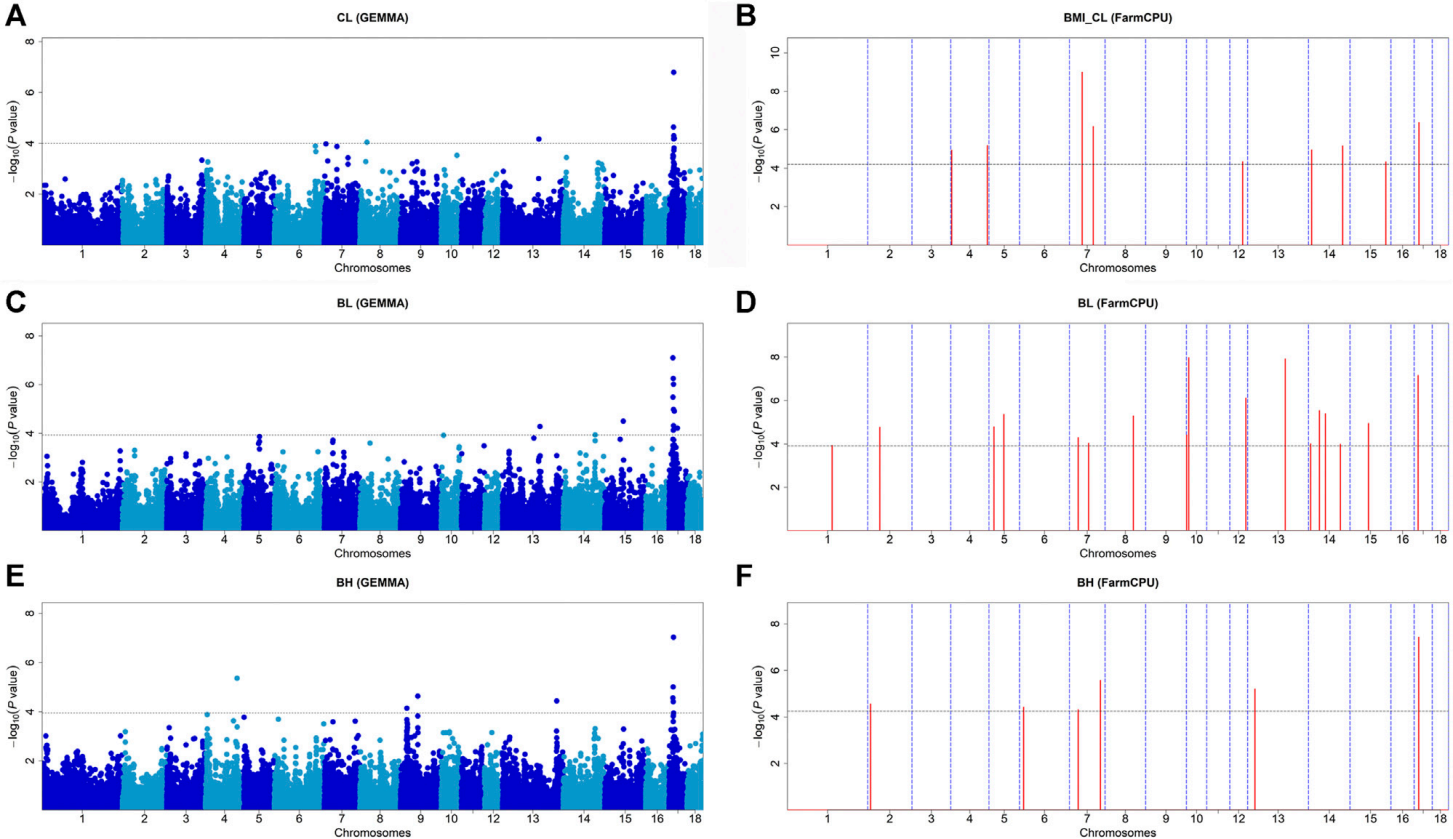
Manhattan plots of GEMMA-based GWAS and FarmCPU-based GWAS for CL, BL, and BH in DLY pigs. **(A,B)** represent the GWAS results conducted by GEMMA-based GWAS (threshold: *p* = 1.00E-04) and FarmCPU-based GWAS (threshold: *p* = 5.05E-05) for CL, respectively. **(C,D)** represent the GWAS results conducted by GEMMA-based GWAS (threshold: *p* = 1.17E-04) and FarmCPU-based GWAS (threshold: *p* = 1.22E-04) for BL, respectively. **(E,F)** represent the GWAS results conducted by GEMMA-based GWAS (threshold: *p* = 1.13E-04) and FarmCPU-based GWAS (threshold: *p* = 5.55E-05) for BH, respectively. The *x*-axis represents the chromosomes, and the *y*-axis represents the -log10 (*p*-value).

Fifteen significant SNPs were found for CL. MLM found seven significant SNPs, and FarmCPU detected nine SNPs. The *WU_10.2_17_17981232* on SSC17 was the most significant SNP identified by GEMMA-based GWAS (*p* = 1.60 × 10^−7^) (Figure 2A) and also identified by FarmCPU GWAS which explained 9.74% of the phenotypic variance (Table 3). The *WU_10.2_7_49907567* on SSC7 was the most significant SNP identified by FarmCPU GWAS (*p* = 1.71 × 10^−8^) (Figure 2B). For BL, the lead SNP was *ALGA0123867* on SSC17, detected by the GEMMA-based GWAS (*p* = 7.91 × 10^−8^). Meanwhile, *ALGA0123867* was the significant SNP detected by both methods. *ALGA0056836* (*p* = 1.07 × 10^−8^) on SSC10 was found by FarmCPU (Figures 2C,D). For BH, there is a significant SNP, *WU_10.2_17_17479009*, on SSC17, which was detected by the two methods. It was also the most significant SNP detected by GEMMA-based GWAS (*p* = 9.23 × 10^−8^) and FarmCPU (*p* = 3.71 × 10^−8^) (Figures 2E,F). The SNPs for BH detected by GEMMA-based GWAS were mainly concentrated on chromosome 17. In contrast, except for one significant SNP, all other SNPs identified by FarmCPU were concentrated on other chromosomes outside SSC17. Among these SNPs, the most significant SNP for BH, namely, *WU_10.2_17_17479009*, was also significantly associated with CL and BL. Meanwhile, *WU_10.2_17_17479009* had a similar effect on these three traits, including CL, BL, and BH. In addition, *DBMA0000205* was significantly associated with CL and BL. *WU_10.2_17_15712448* and *ALGA0123867* were significantly associated with BL and BH.

### Body Mass Index

**FIGURE 3:**
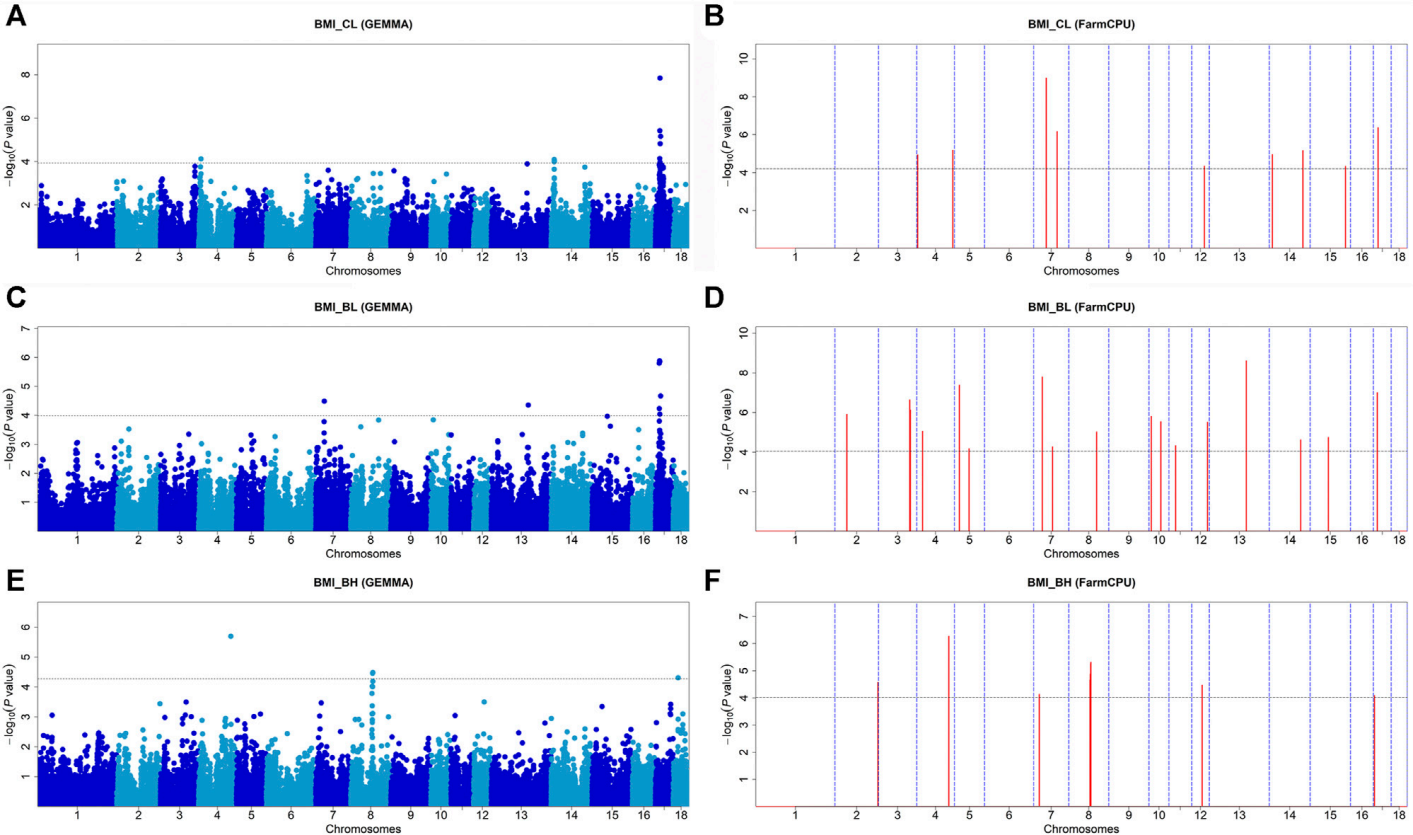
Manhattan plots of GEMMA-based GWAS and FarmCPU-based GWAS for BMI_CL_, BMI_BL_, and BMI_BH_ in DLY pigs. **(A,B)** represent the GWAS results conducted by GEMMA-based GWAS (threshold: *p* = 1.16E-04) and FarmCPU-based GWAS (threshold: *p* = 6.30E-05) for BMI_CL_, respectively. **(C,D)** represent the GWAS results conducted by EMMA-based GWAS (threshold: *p* = 1.04E-04) and FarmCPU-based GWAS (threshold: *p* = 8.85E-05) for BMI_BL_, respectively. **(E,F)** represent the GWAS results conducted by EMMA-based GWAS (threshold: *p* = 5.31E-05) and FarmCPU-based GWAS (threshold: *p* = 9.58E-05) for BMI_BH_, respectively. The *x*-axis represents the chromosomes, and the *y*-axis represents the -log10 (*p*-value).

Seventeen SNPs were significantly associated with BMI_CL_, 22 SNPs with BMI_BL_, and 13 SNPs with BMI_BH_ by these two GWAS methods (Figure 3 and Table 4). GEMMA found 10 significant SNPs for BMI_CL_, 8 significant SNPs for BMI_BL_, and 4 significant SNPs for BMI_BH_. Based on FarmCPU, there were 9 SNPs significantly associated with BMI_CL_, 17 SNPs significantly associated with BMI_BL_, and 12 SNPs significantly associated with BMI_BH_. *WU_10.2_17_17981232* (*p* = 1.42 × 10^−8^) on SSC17 and *WU_10.2_7_49907567* (*p* = 1.02 × 10^−9^) on SSC7 were the most significant SNP identified by GEMMA-based GWAS and FarmCPU GWAS for BMI_CL_ (Figure 3A,B and Table 4), respectively. *WU_10.2_17_17981232* was the only SNP detected by both GWAS methods for BMI_CL_ and explained 10.13% of the phenotypic variance (Table 4). For BMI_BL_, the most significant SNP detected by GEMMA-based GWAS was *WU_10.2_17_15712448* (*p* = 1.30 × 10^−6^) and detected by FarmCPU GWAS was WU_10.2_13_138014916 (*p* = 2.45 × 10^−9^) (Figure 3C,D and Table 4). Among all significant SNPs for BMI_BL_, the explained phenotypic variation of *WU_10.2_17_15712448* was the largest and up to 12.06%. The most significant *SNP* for BMI_BH_ detected by two GWAS methods was *ASGA0022193* (*p* = 2.00 × 10^−6^ from GEMMA-based GWAS; *p* = 5.34 × 10^−7^ from FarmCPU) (Figure 3E,F and Table 4). This site explained 7.49% of the phenotypic variance (Table 4).

### Linkage Disequilibrium Between Significant Single-Nucleotide Polymorphisms

**FIGURE 4:**
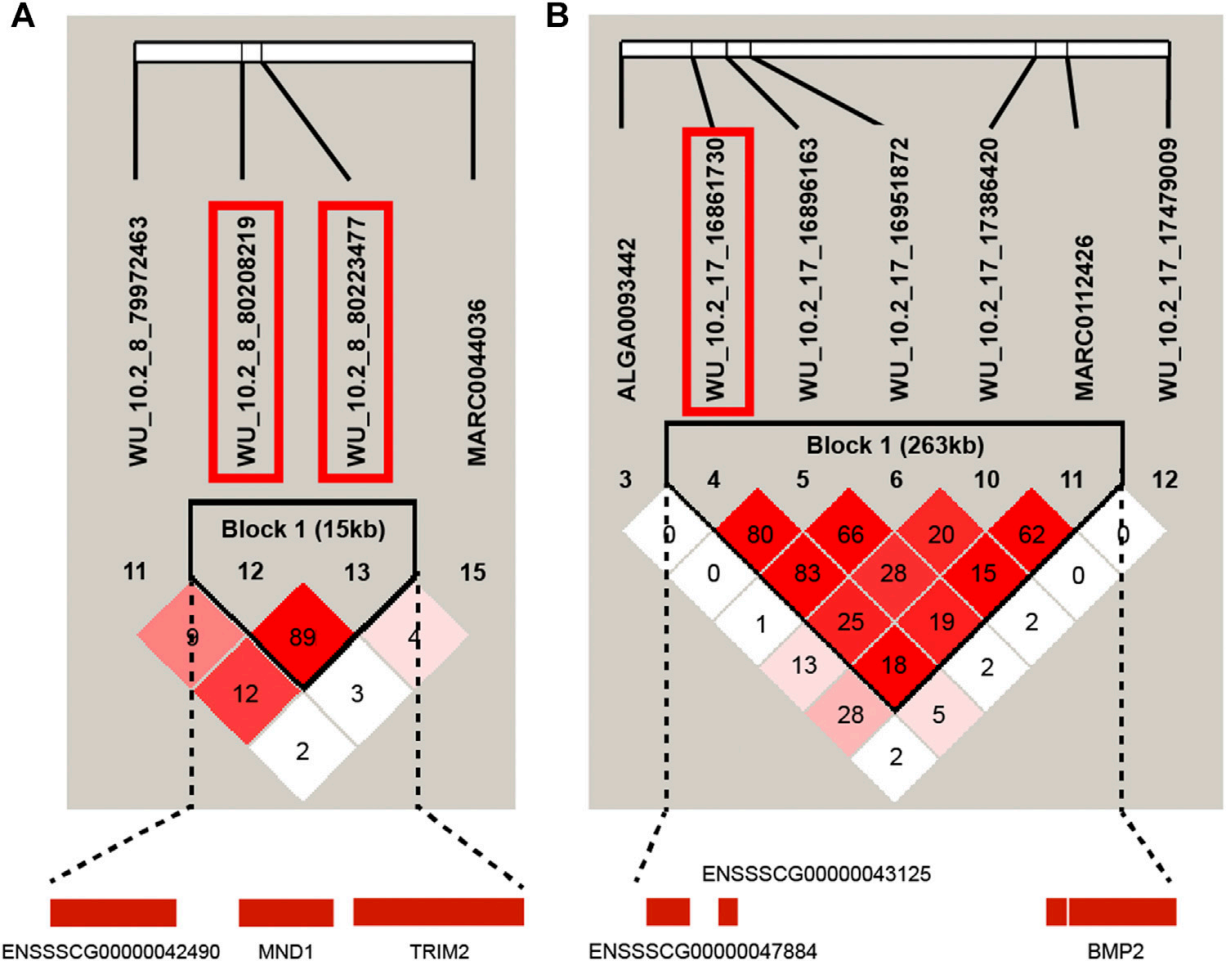
Haplotype blocks for significant SNPs indicate a haplotype block composed of significant SNPs located on SSC8 and SSC17. **(A)** represents the 15-kb LD block in the significant region on SSC8 with two significant SNPs (*WU_10.2_8_80,208,219* and *WU_10.2_8_80223477*) for BMI_BH_. **(B)** is the haplotype block on SSC17 with two significant SNPs (*WU_10.2_17_1,6,861,730* and *WU_10.2_17_1,6,951,872*) for BH.

In this study, the GWAS results showed that some QTL exhibited effects on more than one trait. For instance, *WU_10.2_17_17479009* on SSC17 was significantly associated with CL, BL, BH, BMI_CL_, and BMI_BL_, and *WU_10.2_17_15712448* was significantly associated with BL, BH, and BMI_BL_. We further examined the LD pattern between SNPs in these QTL regions. Two LD block with *r* ^*2*^ > 0.8 respectively distributed on SSC8 and 17 were found. The LD block on SSC8 was 15 kb, including two significant SNPs for BL and BMI_BH_, respectively (Figure 4A). On SSC17, the LD block was 263 kb, which contained one significant SNP for BMI_CL_ (Figure 4B). Interestingly, there are some SNPs on chromosome 17 that are significantly related to CL, BL, BH, and BMI_CL_ and on chromosome 8 for BMI_BH_, such as *WU_10.2_8_80223477* in the LD block on SSC 8 and *WU_10.2_17_17479009* adjacent to the LD block on SSC 17, whether having corrected or uncorrected the BW (Supplementary Table S4), which directly proves that these SNPs are less affected by body weight and have a greater impact on the inquired traits. It is worth noting that the most significant SNP for BH, *WU_10.2_17_17479009*, was adjacent to this block, which also significantly affected CL, BL, BH, BMI_CL_, and BMI_BL_. Interestingly, the *BMP2* gene, the nearest gene of *WU_10.2_17_17479009*, was also located in this 263-kb block. *BMP2*, which has a crucial role in chondrocyte proliferation and maturation, can be considered as one of the candidate genes for body length traits.

## Discussion

Genome-wide association studies provide an opportunity to dissect the genetic architecture of complex traits by leveraging LD between the causative mutations and common SNP markers in pigs (Zhuang et al., 2020). We performed two model-based GWASs on body conformation traits in a DLY pig population, detecting a set of trait-related SNPs, and then based on these SNPs and QTLs, candidate genes were annotated.

### Genetic Loci and Genes for Body Weight

Body weight is a complex quantitative trait in domestic pigs and genomics, and molecular techniques can rapidly advance genetic improvement and increase production levels (Johnson and Nugent, 2003). In this study, four of the seven significant SNPs for BW corresponded to previously discovered QTL after assessment using PigQTLdb (https://www.animalgenome.org). The other three SNPs were newly found in the present study and two of them were identified by FarmCPU. According to two main criteria for listing the lead SNPs (lead signals) and candidate genes (secondary signals), the gene was the nearest to the index SNP, and the gene was found in the vicinity of the lead SNP and was biologically related to the trait (Berndt et al., 2013). We listed the candidate genes in the proximity of these significant SNPs for BW and checked their functions and involved pathways (Supplementary Table S3), all related to BW-relevant phenotypes such as growth, body size, digestive/alimentary system, and skeleton. For example, the *CEL* (Gilham et al., 2007) and *MAP1LC3C* (Patel et al., 2013) genes affecting the digestive/alimentary system of mice may affect the BW. The *HSPG2* on SSC6 was involved in abnormal vertebral segmentation in human and animal models (Turnpenny et al., 2007) and presented different expressions between different genotype individuals at the *VRTN* QTNs, which are causal mutations for thoracic number variation of pig and wild-type pig embryos (Duan et al., 2018). Interestingly, the additional thoracic number will increase the body length and then improve pork production. This suggests that the *HSPG2* gene may influence BW by affecting vertebral development.

### Genetics Loci and Genes for Body Mass Index

Obesity, a growing worldwide health problem, is a complex trait. BMI was commonly employed to assess obesity in human disease study. In humans, BMI was determined by weight and height. In previous studies, BL and BH were used to represent “height” respectively in fat-type pigs, Chinese indigenous Laiwu and Bamaxiang pigs (Zhou et al., 2016; Gong et al., 2019). Nevertheless, limited to the physical structure determined by the pig’s walking style, simply adding the body length and limb height may cause a large error. Hence, we used CL, BL, and BH, respectively, as the “height” to calculate the pig’s BMI value. We found that all the QTLs identified in lean-type pigs, DLY, in this study were not reported previously in fat-type pigs. This suggests that the major effect loci of BMI trait of lean-type pigs and fat-type pigs are different. After comparing pig BMI loci and genes that are nearest to the index SNP of BMIs to these in human (https://www.ncbi.nlm.nih.gov/omim), there was no overlap between pig and human. Among all SNPs associated with these three BMI traits, only *WU_10.2_17_17479009* and *DBMA0000205* were significantly correlated with two or more traits simultaneously, and they had significant effects on both BMI_CL_ and BMI_BL_. The gene nearby *WU_10.2_17_17479009* was bone morphogenetic protein 2 (*BMP2*), and the nearby gene of *DBMA0000205* was phospholipase C Beta 4 (*PLCB4*). These two genes (*BMP2* and *PLCB4*) were related to growth, body size, and skeleton. Moreover, *PLCB4* also affected adipose tissue. Although the other SNPs are different, the function of genes nearest to the significant SNPs of three kinds of BMIs was similar. *TGFBR3*, *BNIP3L*, and *PLCB4* were closest to the significant SNPs of BMI_CL_. *OTOG*, *ITPR3*, *ITPR3*, and *RASSF2* were nearest to significant SNPs of BMI_BL_. *FRAS1,* and *FBXW7* were closest to significant SNPs of BMI_BH_. These genes all affect the adipose tissue, growth, body size, skeleton or nervous system (Ignatieva et al., 2016), and digestive/alimentary system of mice.

The BMI of a pig can be considered as meat yield ability in specific body length. The higher the BMI value, the stronger the pig’s potential meat yield for the pigs of the same age and under the same feeding environment. However, the BMIs of pigs at different ages are significantly different, and the corresponding significant SNPs were also different (Zhou et al., 2016). Considering that major genes affecting this trait may vary with the age of pigs, more genetic analyses of pig BMI are needed to discover BMI-related loci and their underlying mechanisms.

### Genetic Loci and Genes for Carcass Length, Body Length, and Body Height

Among 53 significant SNPs that are associated with CL, BL, and BH traits based on two GWAS models, five SNPs (*ALGA0123867*, *DBMA0000205*, *SIRI0000046*, *WU_10.2_17_15712448*, and *WU_10.2_17_17479009*) were identified to be significantly associated with more than one trait. The candidate genes closest to these five SNPs were *PRNP*, *PLCB4*, *ITPR3*, *SHLD1*, and *BMP2*. These genes all influence skeletal growth and body size in mice. We further aggregated evidence from our data, human (https://www.ncbi.nlm.nih.gov/omim/) and mouse genetic databases (http://www.informatics.jax.org/), and proposed the candidate genes based on their functional relevance to traits. A number of genes, such as *DAGLA*, *RASSF2*, *MMUT*, *PEBP4*, *NR4A2*, *FERMT1*, *SOCS6*, *TBX3*, *TGFBR3*, *TRAPPC9*, *UCHL5*, *VAC14*, *WNT2B*, *GRM5*, *ULK4*, and *SCN8A* affect growth, body size, and skeleton in mice. Interestingly, among these genes, *EGLN3*, *KSR1*, and *GRM5* were involved in the growth of adipose tissue. This hints that these genes may affect the two traits simultaneously or influence body length traits by changing the backfat thickness or conversely.

The most strongly associated variant was often located near the causal genes (Lango Allen et al., 2010). Nearly half of the significant SNPs for CL, BL, and BH traits were on SSC17. Among these SNPs, only *WU_10.2_17_17479009* showed significant effects on multiple carcass traits, including CL, BL, and BH, even BMI_CL_ and BMI_BL_. Considering these facts, we postulate that one or more causative genes exist there that regulate the pathway involving the growth and carcass traits of pigs. The most likely candidate gene is *BMP2*, which was nearest to *WU_10.2_17_17479009.* Thus, gene encodes a secreted ligand of the TGF-beta superfamily of proteins, which involves transforming the growth factor-beta (TGF-beta) signaling pathway, playing a role in bone and cartilage development (Blaj et al., 2018). The *BMP2* conditional knockout (cKO) mice showed smaller calvaria, thoracic cavities, and shorter spines and hind limbs than Cre-negative littermates (Shu et al., 2011). In humans, the heterozygous mutation in the *BMP2* gene resulted in short stature, facial dysmorphism, and skeletal anomalies (Tan et al., 2017). Although there is no direct evidence in pigs suggesting *BMP2* as a causal gene of pig body size traits, some studies have shown that some mutation sites in or near this gene were associated with body length, body depth, and body width (Fan et al., 2011). Then, we conducted a gene enrichment analysis using candidate genes including the closest genes and those within the 1-Mb region of significant SNPs for body conformation traits to have a comprehensive understanding of their signaling pathways involved in regulating body size (Supplementary Table S5). The top 20 GO-enriched terms included cell growth, negative regulation of cell population proliferation, and chondrocyte differentiation, which are closely related to body conformation. Of note, *BMP2* was in two of these three pathways. This result provided further evidence that *BMP2* was the most probable candidate gene for body conformation traits. Although further studies are needed to dissect the genetic architecture of body conformation traits, our findings have identified a number of novel loci pinpointing biologically relevant genes and pathways for body weights and conformations.

## Conclusion

In this study, we identified 82 SNPs associated with seven body conformation traits in DLY pigs using GEMMA-based GWAS and FarmCPU-based GWAS. We then identified three genomic regions and several genes related to body conformation traits in pigs. *WU_10.2_17_17479009* was the only SNP that affected more than three traits and showed pleiotropic effects on CL, BL, BH, BMI_CL_, and BMI_BL_ in pigs. Specifically, the *BMP2* gene is proposed as a strong candidate gene for body size due to the effect on CL, BL, BH, BMI_CL_, and BMI_BL_ and is involved in growth and bone development. In addition, we expect that our results provide a comprehensive understanding of the BMI trait, which has not been studied adequately in pigs. Altogether, this study not only benefits the molecular breeding for body conformation-related traits of the DLY pig but also advances our knowledge of the poorly understood of genetic loci or genes controlling BMI in pigs.

## Data Availability

All genotypic data were deposited in Figshare https://doi.org/10.6084/m9.figshare.16692094.v1.

## Abbreviations

BCT, body conformation traits; BMI, body mass index; BW, body weight; BL, body length; BH, body height; BMICL, body mass index (weight/height2, CL be height); BMIBL, body mass index (weight/height2, BL be height); BMIBH, body mass index (weight/height2, BH be height); CL, carcass length; GWAS, genome-wide association studies; SNP, single-nucleotide polymorphism.
