# Supplementary material for: Genome-Wide Detection of Genetic Loci and Candidate Genes for Body Conformation Traits in Duroc × Landrace × Yorkshire Crossbred Pigs
Source: Front Genet. 2021 Oct 11;12:664343. doi: 10.3389/fgene.2021.664343 (PMC8542986; doi:10.3389/fgene.2021.664343)
Supplement: Supplementary file 1 [file DataSheet1.docx]

## Supplementary information

**Figure S1.** The difference in "height" of human and pig. (A) The height of human. (B) The ideal height of pig. (C) The body length and body height of pigs were treated as "height".

**Figure S2.** Genetic structure of DLY pigs. (A) Plot of the ﬁrst two principal components of 311 pigs. (B) Scree plot showing the selection of principal components for GWAS. (C) LD decay across the whole genome of the association panel. The red dotted line represents the LD threshold for the association panel (*r*^2^ = 0.1)

**Figure S3.** Quantile-quantile (Q-Q) plots of single-locus GWAS for body weight and body conformation traits in DLY pigs. Q-Q plots show the observed versus expected negative log 10 *P*-values.

**Table S1**. Phenotypic correlation coefficients between body weight and body conformation traits.

**Table S2**. Phenotypic correlation coefficients among BMI traits.

**Table S3**. The significant SNPs associated genes for BW, CSL, BL, BH, BMI_CSL_, BMI_BL_ and BMI_BH_ identified in the GWAS. Genes nearby the region of 1 Mb of every variant are listed with chromosome and cluster numbers.

**Table S4.** The common significant SNPs associated genes for BW, CL, BL, BH, BMI_CL_, BMI_BL_ and BMI_BH_ after uncorrected and corrected BW.

**Table S5.** Top 20 clusters with their representative enriched terms.


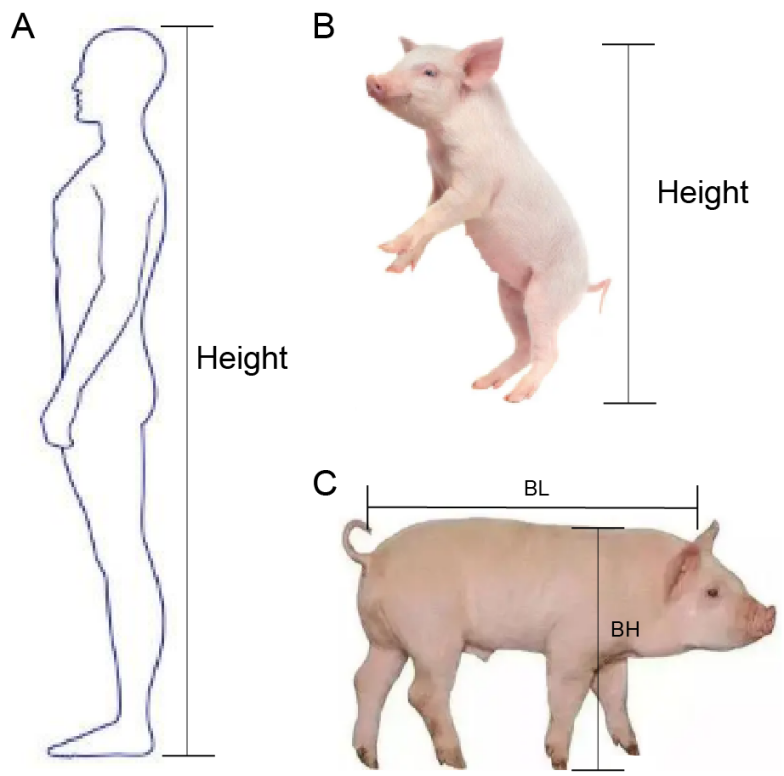


**Figure S1.** The difference in "height" of human and pig. (A) The height of human. (B) The ideal height of pig. (C) The body length and body height of pigs were treated as "height".


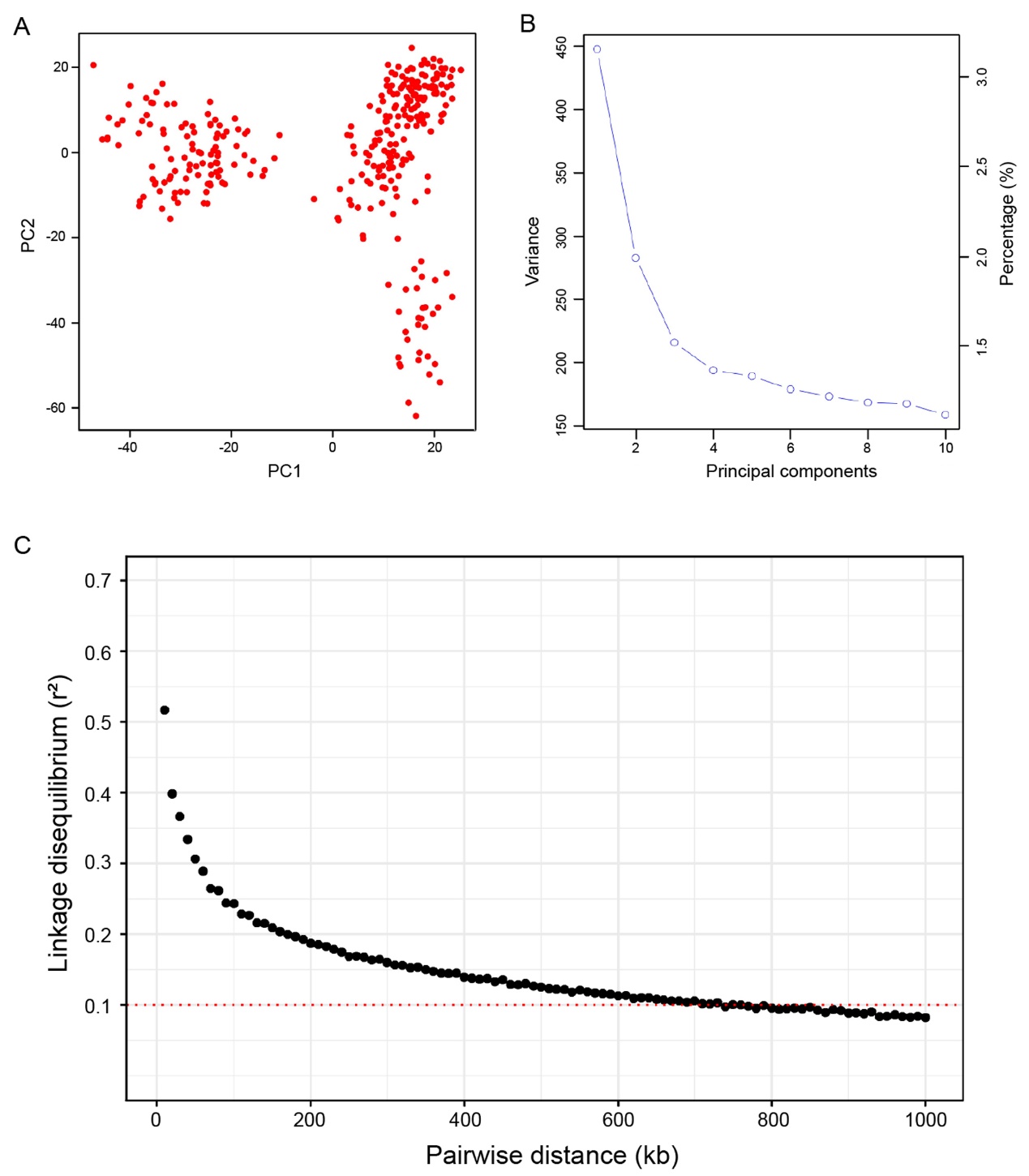


**Figure S2.** Genetic structure of DLY pigs. (A) Plot of the ﬁrst two principal components of 311 pigs. (B) Scree plot showing the selection of principal components for GWAS. (C) LD decay across the whole genome of the association panel. The red dotted line represents the LD threshold for the association panel (*r*^2^ = 0.1)


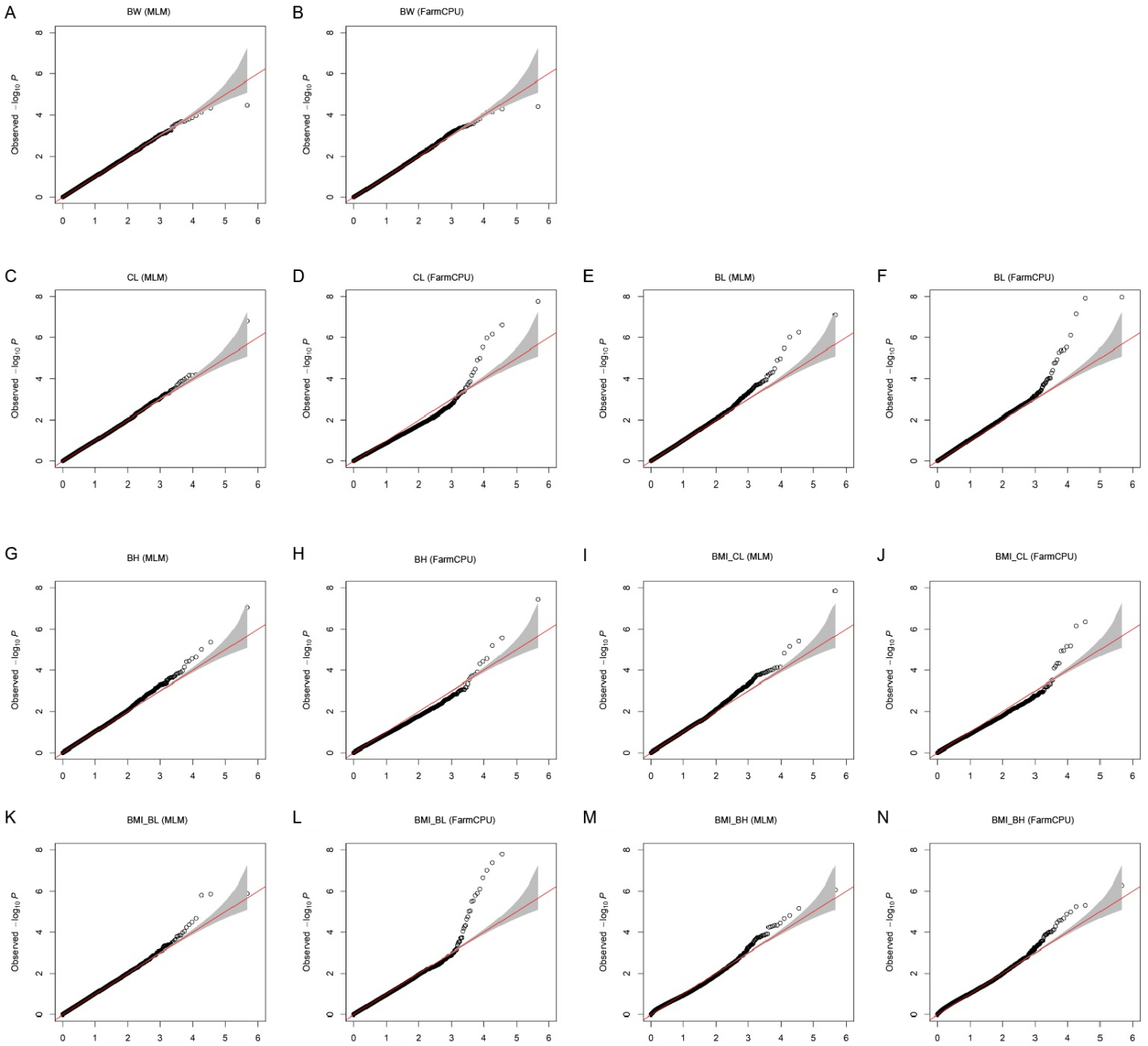


**Figure S3.** Quantile-quantile (Q-Q) plots of single-locus GWAS for body weight and body conformation traits in DLY pigs. Q-Q plots show the observed versus expected negative log 10 *P*-values.

Table S1. Phenotypic correlation coefficients between body weight and body conformation traits.

| Traits | BW | BL | BH | CL |
| --- | --- | --- | --- | --- |
| BW | 1 |  |  |  |
| BL | 0.55** | 1 |  |  |
| BH | 0.36** | 0.58** | 1 |  |
| CL | 0.31** | 0.53** | 0.35** | 1 |

Table S2. Phenotypic correlation coefficients among BMI traits.

| Traits | BMI_BL_ | BMI_BH_ | BMI_CL_ |
| --- | --- | --- | --- |
| BMI_BL_ | 1 |  |  |
| BMI_BH_ | 0.81** | 1 |  |
| BMI_CL_ | 0.73** | 0.69** | 1 |

Table S3. The significant SNPs associated genes for BW, CL, BL, BH, BMI_CL_, BMI_BL_ and BMI_BH_ identified in the GWAS. Genes nearby the region of 1 Mb of every variant are listed with chromosome and cluster numbers.

| Trait | SSC^a^ | SNP ID | Position (bp)^b^ | Genes within the 1 Mb region |
| --- | --- | --- | --- | --- |
| BMI_CL_ | 4 | WU_10.2_4_3046732 | 3559394 | TRAPPC9 KCNK9 |
|  | 4 | ASGA0017873 | 7875059 | ST3GAL1 NDRG1 CCN4 TG |
|  | 4 | WU_10.2_4_136884741 | 125301443 | GLMN C1orf146 BTBD8 EPHX4 BRDT TGFBR3 CDC7 HFM1 ZNF644 |
|  | 7 | WU_10.2_7_49907567 | 43478519 | CENPQ GLYATL3 RHAG CRISP2 CRISP3 CRISP1 DEFB133 |
|  | 7 | ASGA0034652 | 81310017 | RYR3 ARRDC4 |
|  | 12 | ALGA0122685 | 43952687 | RAB11FIP4 NF1 EVI2B OMG WSB1 KSR1 NOS2 NLK |
|  | 14 | DIAS0004697 | 10274713 | EBF2 PPP2R2A BNIP3L PNMA2 DPYSL2 ADRA1A |
|  | 14 | ASGA0061212 | 10327418 | EBF2 PPP2R2A BNIP3L PNMA2 DPYSL2 ADRA2A |
|  | 14 | 14_12070780 | 10892893 | PNMA2 DPYSL2 ADRA2A STMN4 TRIM35 PTK2B CHRNA2 CLU SCARA3 |
|  | 14 | ASGA0066313 | 116729015 | -- |
|  | 15 | WU_10.2_15_136106170 | 122789034 | -- |
|  | 17 | WU_10.2_17_16861730 | 15492508 | FERMT1 BMP2 |
|  | 17 | WU_10.2_17_17479009 | 15827454 | BMP2 |
|  | 17 | WU_10.2_17_17981232 | 16253154 | HAO1 BMP2 |
|  | 17 | MARC0028591 | 16634316 | HAO1 PLCB1 |
|  | 17 | ALGA0093478 | 16919581 | HAO1 PLCB2 |
|  | 17 | DBMA0000205 | 18319097 | PLCB4 LAMP5 PAK5 |
| BMI_BL_ | 2 | M1GA0024370 | 41570652 | SAAL1 SERGEF KCNC1 OTOG USH1C ABCC8 KCNJ11 PIK3C2A |
|  | 3 | ALGA0020800 | 108685228 | CAPN14 CAPN13 YPEL5 |
|  | 3 | WU_10.2_3_117349436 | 110594886 | CLIP4 PCARE WDR43 TRMT61B SPDYA PPP1CB PLB1 FOSL2 BABAM2 |
|  | 4 | ALGA0023916 | 20318986 | SAMD12 EXT1 |
|  | 5 | H3GA0015868 | 17192568 | TMPRSS12 SLC4A8 SCN8A ACVRL1 ACVR1B GRASP NR4A1 ATG101 KRT80 KRT7 KRT85 |
|  | 5 | ALGA0031952 | 50860053 | ETNK1 C2CD5 ST8SIA1 |
|  | 7 | SIRI0000046 | 29878705 | COL21A1 VPS52 B3GALT4 WDR46 PFDN6 ZBTB22 DAXX KIFC1 PHF1 CUTA SYNGAP1 ITPR3 IP6K3 LEMD2 GRM4 |
|  | 7 | ASGA0034397 | 65649418 | EAPP EGLN3 NPAS3 |
|  | 8 | ALGA0124320 | 96665022 | SCLT1 JADE1 LARP1B ABHD18 MFSD8 PLK4 SLC25A31 INTU |
|  | 10 | ALGA0056836 | 7925254 | GPATCH2 SPATA17 RRP15 TGFB2 |
|  | 10 | ALGA0106806 | 41215023 | MAP3K8 MTPAP JCAD SVIL ZNF438 |
|  | 11 | ALGA0061436 | 23388539 | SMIM2 ENOX1 |
|  | 12 | WU_10.2_12_57752831 | 55009055 | STX8 CFAP52 USP43 DHRS7C GSG1L2 GLP2R RCVRN MYH13 MYH8 MYH3 SCO1 ADPRM TMEM220 |
|  | 13 | WU_10.2_13_138014916 | 128619134 | CCDC50 GMNC UTS2B |
|  | 14 | ALGA0080935 | 108956441 | SLIT1 FRAT1 FRAT2 RRP12 PGAM1 EXOSC1 ZDHHC16 MMS19 UBTD1 ANKRD2 C10orf62 MORN4 AVPI1 MARVELD1 ZFYVE27 GOLGA7B CRTAC1 |
|  | 15 | ALGA0085736 | 63541092 | NR4A2 GPD2 |
|  | 17 | ALGA0123867 | 13717308 | PSD3 PRNP PRND RASSF2 SLC23A2 CDS2 |
|  | 17 | WU_10.2_17_14580447 | 13779206 | PRNP PRND RASSF2 SLC23A2 CDS2 PROKR2 |
|  | 17 | WU_10.2_17_15712448 | 14734253 | PROKR2 GPCPD1 SHLD1 TRMT6 CRLS1 FERMT1 |
|  | 17 | WU_10.2_17_17075196 | 15689085 | BMP2 |
|  | 17 | WU_10.2_17_17479009 | 15827454 | BMP2 |
|  | 17 | DBMA0000205 | 18319097 | PLCB4 LAMP5 PAK5 |
| BMI_BH_ | 2 | MARC0035424 | 149466993 | JAKMIP2 SPINK1 SCGB3A2 C5orf46 SPINK5 SPINK14 SPINK6 SPINK7 SPINK9 FBXO38 |
|  | 4 | ASGA0022193 | 111702541 | STXBP3 FNDC7 HENMT1 FAM102B VAV3 |
|  | 7 | ASGA0031627 | 19251635 | NRSN1 DCDC2 MRS2 GPLD1 KIAA0319 ACOT13 C6orf62 GMNN RIPOR2 ARMH2 |
|  | 8 | MARC0065833 | 76712691 | ARFIP1 TMEM154 FBXW7 GATB |
|  | 8 | WU_10.2_8_80223477 | 75732460 | TLR2 TMEM131L MND1 TRIM2 FHDC1 ARFIP1 |
|  | 8 | H3GA0025014 | 76555090 | FHDC1 ARFIP1 TMEM154 FBXW7 |
|  | 8 | ASGA0039051 | 74641551 | RBM46 LRAT FGG FGA DCHS2 |
|  | 8 | MARC0109188 | 73439961 | CNOT6L FRAS1 |
|  | 8 | WU_10.2_8_80208219 | 75717201 | TLR2 TMEM131L MND1 TRIM2 FHDC1 ARFIP1 |
|  | 8 | ALGA0048253 | 74373748 | FRAS1 NPY2R RBM46 LRAT FGG FGA DCHS2 |
|  | 12 | ASGA0054390 | 36455949 | VMP1 TUBD1 RNFT1 MED13 INTS2 BRIP1 BCAS3 |
|  | 17 | ALGA0092770 | 4018290 | MSR1 |
|  | 18 | WU_10.2_18_18355722 | 17275733 | PLXNA4 MKLN1 |
| BW | 1 | ASGA0001774 | 26631978 | ARFGEF3 TNFAIP3 OLIG3 IFNGR1 IL22RA2 |
|  | 1 | ASGA0005703 | 215031222 | KDM4C |
|  | 1 | WU_10.2_1_306708221 | 272782419 | CFAP77 BARHL1 DDX31 GTF3C4 AK8 SPACA9 TSC1 GFI1B CEL RPL7A GBGT1 SURF6 SURF1 SURF2 SURF4 STKLD1 REXO4 ADAMTS13 CACFD1 SLC2A6 MYMK ADAMTSL2 FAM163B DBH SARDH MED22 |
|  | 3 | ALGA0021159 | 115678132 | -- |
|  | 6 | M1GA0008725 | 80122519 | ALPL RAP1GAP USP48 LDLRAD2 HSPG2 ZBTB40 EPHA8 C1QA C1QC C1QB |
|  | 6 | WU_10.2_6_135404715 | 146999505 | PDE4B LEPR DNAJC6 AK4 JAK1 |
|  | 10 | ASGA0091894 | 15367300 | CDC42BPA AHCTF1 EXO1 MAP1LC3C BECN2 PLD5 |
| BL | 1 | WU_10.2_1_168922259 | 152527914 | SOCS6 RTTN CD226 DOK6 |
|  | 2 | M1GA0024370 | 41570652 | SAAL1 SERGEF KCNC1 OTOG USH1C ABCC8 KCNJ11 PIK3C2A |
|  | 5 | H3GA0015868 | 17192568 | TMPRSS12 SLC4A8 SCN8A ACVRL1 ACVR1B GRASP NR4A1 ATG101 KRT80 KRT7 KRT85 |
|  | 5 | ALGA0031952 | 50860053 | ETNK1 C2CD5 ST8SIA1 |
|  | 7 | SIRI0000046 | 29878705 | COL21A1 VPS52 B3GALT4 WDR46 PFDN6 ZBTB22 DAXX KIFC1 PHF1 CUTA SYNGAP1 ITPR3 IP6K3 LEMD2 GRM4 |
|  | 7 | ALGA0042427 | 65595703 | EAPP EGLN3 NPAS3 |
|  | 7 | ASGA0034393 | 65625414 | EAPP EGLN3 NPAS3 |
|  | 8 | ALGA0124320 | 96665022 | SCLT1 JADE1 LARP1B ABHD18 MFSD8 PLK4 SLC25A31 INTU |
|  | 10 | ASGA0045707 | 740406 | CDC73 B3GALT2 RO60 RGS13 RGS1 |
|  | 10 | ALGA0056836 | 7925254 | GPATCH2 SPATA17 RRP15 TGFB2 |
|  | 12 | WU_10.2_12_57752831 | 55009055 | STX8 CFAP52 USP43 DHRS7C GSG1L2 GLP2R RCVRN MYH13 MYH8 MYH3 SCO1 ADPRM TMEM220 |
|  | 13 | WU_10.2_13_138014916 | 128619134 | GMNC UTS2B CCDC50 |
|  | 14 | ASGA0060896 | 7192572 | SLC39A14 SORBS3 BIN3 EGR3 PEBP4 CHMP7 LOXL2 ENTPD4 SLC25A37 |
|  | 14 | ASGA0062769 | 37378781 | TBX3 TBX5 |
|  | 14 | ALGA0077889 | 57422121 | SLC35F3 KCNK1 MAP3K21 NTPCR |
|  | 14 | ALGA0080935 | 108956441 | SLIT1 FRAT1 FRAT2 RRP12 PGAM1 EXOSC1 ZDHHC16 MMS19 UBTD1 ANKRD2 C10orf62 MORN4 AVPI1 MARVELD1 ZFYVE27 GOLGA7B CRTAC1 |
|  | 15 | ALGA0085736 | 63541092 | NR4A2 GPD2 |
|  | 17 | ALGA0123867 | 13717308 | PSD3 PRNP PRND RASSF2 SLC23A2 CDS2 |
|  | 17 | WU_10.2_17_14580447 | 13779206 | PRNP PRND RASSF2 SLC23A2 CDS2 PROKR2 |
|  | 17 | WU_10.2_17_15712448 | 14734253 | PROKR2 GPCPD1 SHLD1 TRMT6 CRLS1 FERMT1 |
|  | 17 | ASGA0075536 | 15196027 | SHLD1 TRMT6 CRLS1 FERMT1 |
|  | 17 | WU_10.2_17_17075196 | 15689085 | BMP2 |
|  | 17 | WU_10.2_17_17013787 | 15710331 | BMP2 |
|  | 17 | WU_10.2_17_17479009 | 15827454 | BMP2 |
|  | 17 | DBMA0000205 | 18319097 | PLCB4 LAMP5 PAK5 |
|  | 17 | DRGA0016692 | 29138019 | RALGAPA2 KIZ XRN2 NKX2-4 NKX2-2 PAX1 |
| BH | 2 | WU_10.2_2_9315312 | 9766370 | SCGB1A1 ASRGL1 INCENP BEST1 RAB3IL1 FADS3 FEN1 DAGLA SYT7 LRRC10B SDHAF2 CPSF7 TMEM216 TMEM138 CYB561A3 TKFC DDB1 VWCE |
|  | 4 | MARC0012235 | 107879743 | LRIG2 SLC16A1 PPM1J MOV10 ST7L WNT2B CTTNBP2NL |
|  | 6 | WU_10.2_6_13750573 | 13837643 | ST3GAL2 FCSK SF3B3 IL34 MTSS2 VAC14 CMTR2 |
|  | 7 | SIRI0000046 | 29878705 | COL21A1 VPS52 B3GALT4 WDR46 PFDN6 ZBTB22 DAXX KIFC1 PHF1 CUTA SYNGAP1 ITPR3 IP6K3 LEMD2 GRM4 |
|  | 7 | ALGA0044383 | 105954725 | -- |
|  | 9 | ALGA0112140 | 21921748 | RAB38 CTSC GRM5 |
|  | 9 | H3GA0027617 | 59226678 | NTM OPCML |
|  | 13 | WU_10.2_13_27755688 | 25296621 | CTNNB1 ULK4 TRAK1 |
|  | 13 | ALGA0073322 | 185848189 | NCAM2 |
|  | 17 | ALGA0123867 | 13717308 | PSD3 PRNP PRND RASSF2 SLC23A2 CDS2 |
|  | 17 | WU_10.2_17_15792357 | 14642640 | CDS2 PROKR2 GPCPD1 SHLD1 TRMT6 CRLS1 FERMT1 |
|  | 17 | WU_10.2_17_15712448 | 14734253 | PROKR2 GPCPD1 SHLD1 TRMT6 CRLS1 FERMT1 |
|  | 17 | WU_10.2_17_17479009 | 15827454 | BMP2 |
|  | 17 | DRGA0016582 | 15949323 | BMP2 |
| CL | 3 | MARC0004652 | 6498219 | TRRAP SMURF1 KPNA7 ARPC1B PDAP1 BUD31 CPSF4 ZNF394 ZKSCAN5 ZNF789 FAM200A ZSCAN25 FOPNL |
|  | 4 | WU_10.2_4_3046732 | 3559394 | TRAPPC9 KCNK9 |
|  | 4 | WU_10.2_4_136884741 | 125301443 | GLMN C1orf146 BTBD8 EPHX4 BRDT TGFBR3 CDC7 HFM1 ZNF644 |
|  | 7 | WU_10.2_7_49907567 | 43478519 | CENPQ GLYATL3 RHAG CRISP2 CRISP3 CRISP1 DEFB133 |
|  | 7 | ASGA0034652 | 81310017 | RYR3 ARRDC4 |
|  | 8 | WU_10.2_8_25141199 | 24043163 | -- |
|  | 12 | ALGA0122685 | 43952687 | RAB11FIP4 NF1 EVI2B OMG WSB1 KSR1 NOS2 NLK |
|  | 13 | ALGA0067792 | 8080882 | ZNF385D |
|  | 13 | WU_10.2_13_134401849 | 124927734 | AHSG FETUB KNG1 EIF4A2 RFC4 ST6GAL1 RTP1 MASP1 RTP2 BCL6 |
|  | 14 | 14_12070780 | 10892893 | PNMA2 DPYSL2 ADRA2A STMN4 TRIM35 PTK2B CHRNA2 CLU SCARA3 |
|  | 17 | WU_10.2_17_17479009 | 15827454 | BMP2 |
|  | 17 | WU_10.2_17_17981232 | 16253154 | BMP2 HAO1 |
|  | 17 | WU_10.2_17_18300615 | 16401737 | HAO1 |
|  | 17 | MARC0028591 | 16634316 | HAO1 PLCB4 |
|  | 17 | DBMA0000205 | 18319097 | PLCB4 LAMP5 PAK5 |

**Table S4.** The common significant SNPs associated genes for BW, CL, BL, BH, BMI_CL_, BMI_BL_ and BMI_BH_ after uncorrected and corrected BW.

| **Trait** | **SSCa** | **SNP ID** | **Position (bp)b** | **MAF** | **Uncorrected** | | | **Corrected** | | | **Distance(bp)** | **Nearest gene** |
| --- | --- | --- | --- | --- | --- | --- | --- | --- | --- | --- | --- | --- |
|  |  |  |  |  | **P-value (MLM)** | **P-value (FarmCPU)** | **r2/%c** | **P-value (MLM)** | **P-value (FarmCPU)** | **r2/%c** |  |  |
| **CL** | 17 | *WU_10.2_17_17981232* | 16253154 | 0.33 | 1.60E-07 | 4.92E-07 | 8.9 | 1.60E-07 | 2.94E-06 | 9.74 | 491286 | *HAO1* |
| **BL** | 10 | *ALGA0056836* | 7925254 | 0.33 |  | 9.89E-07 | 6.37 |  | 1.07E-08 | 4.94 | within | *SPATA17* |
|  | 17 | *ALGA0123867* | 13717308 | 0.26 | 7.91E-08 | 3.99E-05 | 14.06 | 7.91E-08 | 6.96E-08 | 13.04 | 11990 | *PRNP* |
| **BH** | 13 | *WU_10.2_13_27755688* | 25296621 | 0.23 |  | 1.08E-05 | 5.77 |  | 6.25E-06 | 3.39 | within | *ULK4* |
|  | 17 | *WU_10.2_17_17479009* | 15827454 | 0.37 | 9.23E-08 | 1.54E-09 | 8.51 | 9.23E-08 | 3.71E-08 | 9.5 | 66239 | *BMP2* |
| **BMI_CL_** | 17 | *WU_10.2_17_17981232* | 16253154 | 0.33 | 1.84E-05 | 8.25E-05 | 4.72 | 1.42E-08 | 4.32E-07 | 10.13 | 491286 | *HAO1* |
| **BMI_BL_** | 7 | *SIRI0000046* | 29878705 | 0.12 | 1.07E-04 |  | 3.05 | 3.25E-05 | 1.62E-08 | 5.9 | 23455 | *ITPR3* |
|  | 13 | *WU_10.2_13_138014916* | 128619134 | 0.35 | 9.10E-06 | 1.03E-05 | 8.42 | 4.39E-05 | 2.45E-09 | 7.5 | 238 | *CCDC50* |
| **BMI_BH_** | 2 | *MARC0035424* | 149466993 | 0.09 | 6.95E-06 | 2.28E-05 | 5.07 |  | 2.66E-05 | 4.22 | 717 | *SPINK6* |
|  | 4 | *ASGA0022193* | 111702541 | 0.08 | 8.64E-07 | 4.03E-07 | 7.36 | 2.00E-06 | 5.34E-07 | 7.49 | 68270 | *SLC25A24* |
|  | 8 | *MARC0109188* | 73439961 | 0.1 | 5.49E-05 | 2.44E-05 | 4.53 |  | 2.15E-05 | 4.29 | 62703 | *FRAS1* |
|  | 8 | *ALGA0048253* | 74373748 | 0.09 | 3.59E-05 | 6.41E-05 | 3.86 |  | 5.30E-05 | 3.55 | 8768 | *RBM46* |
|  | 8 | *ASGA0039051* | 74641551 | 0.1 | 2.17E-05 | 1.44E-05 | 4.72 |  | 1.32E-05 | 4.4 | 23047 | *DCHS2* |
|  | 8 | *WU_10.2_8_80208219* | 75717201 | 0.12 | 5.24E-05 | 2.52E-05 | 5.09 |  | 2.72E-05 | 5 | 27356 | *MND1* |
|  | 8 | *WU_10.2_8_80223477* | 75732460 | 0.1 | 1.55E-05 | 5.68E-06 | 6.01 | 3.46E-05 | 5.69E-06 | 5.69 | within | *MND1* |
|  | 8 | *H3GA0025014* | 76555090 | 0.11 |  | 1.16E-05 | 4.91 |  | 1.04E-05 | 5.22 | 73068 | *FBXW7* |
|  | 8 | *MARC0065833* | 76712691 | 0.1 | 4.75E-05 | 6.20E-06 | 5.07 | 3.27E-05 | 4.92E-06 | 5.7 | 13340 | *FBXW7* |
|  | 12 | *ASGA0054390* | 36455949 | 0.11 | 4.65E-05 | 2.76E-05 | 4.98 |  | 3.40E-05 | 4.38 | 12252 | *BRIP1* |

Table S5. Top 20 clusters with their representative enriched terms.

| No. | Category | Term | Description | Log(P) | Count | Gene Symbols |
| --- | --- | --- | --- | --- | --- | --- |
| 1 | KEGG Pathway | hsa05142 | Chagas disease (American trypanosomiasis) | -7.73 | 12 | C1QA,C1QB,C1QC,IFNGR1,KNG1,NOS2,PLCB2,PLCB4,PPP2R2A,TGFB2,TLR2,PLCB1 |
| 2 | GO Biological Processes | GO:1901214 | regulation of neuron death | -6.19 | 17 | C1QA,CLU,CTNNB1,DAXX,PTK2B,GRM4,NF1,NR4A2,PRNP,TGFB2,TSC1,SYNGAP1,SLC23A2,TRIM2,FBXW7,BARHL1,EGLN3 |
| 3 | GO Biological Processes | GO:0030155 | regulation of cell adhesion | -5.72 | 27 | ACVRL1,BCL6,BMP2,MAP3K8,EGR3,EPHA8,PTK2B,FGA,FGG,JAK1,KNG1,NF1,NPY2R,PPP1CB,PRNP,TGFB2,TSC1,SCGB1A1,SOCS6,RIPOR2,VAV3,SPINK5,GLMN,TMEM131L,BCAS3,FERMT1,PLXNA4 |
| 4 | CORUM | CORUM:6418 | C1q complex | -5.64 | 3 | C1QA,C1QB,C1QC |
| 5 | GO Biological Processes | GO:1905114 | cell surface receptor signaling pathway involved in cell-cell signaling | -5.59 | 24 | BMP2,CHRNA2,CTNNB1,EXT1,PTK2B,MOV10,NPY2R,NR4A2,PLCB2,TLR2,TNFAIP3,WNT2B,CCN4,FRAT1,PLCB1,TMEM131L,FRAT2,DCDC2,NLK,SDHAF2,FERMT1,SMURF1,CDC73,JADE1 |
| 6 | GO Biological Processes | GO:0051347 | positive regulation of transferase activity | -5.32 | 25 | ADRA1A,ADRA2A,BMP2,CLU,MAP3K8,DAXX,EPHA8,PTK2B,GRM4,GRM5,INCENP,PRNP,RFC4,TGFB2,KSR1,RASSF2,VAV3,FBXW7,VAC14,PAK5,AVPI1,MAP3K21,ARRDC4,IL34,SPDYA |
| 7 | WikiPathways | WP1424 | Globo Sphingolipid Metabolism | -5.18 | 5 | ST6GAL1,ST3GAL1,ST3GAL2,ST8SIA1,GBGT1 |
| 8 | GO Biological Processes | GO:0090066 | regulation of anatomical structure size | -4.97 | 20 | ADRA1A,ADRA2A,DBH,DPYSL2,EXT1,PTK2B,FGA,FGG,KNG1,PIK3C2A,SVIL,TSC1,KIAA0319,USH1C,ARPC1B,VAV3,ARFIP1,PLXNA4,ZFYVE27,UTS2B |
| 9 | GO Biological Processes | GO:0090087 | regulation of peptide transport | -4.97 | 22 | ADRA2A,BNIP3L,FGA,FGG,GPLD1,ITPR3,KCNJ11,NOS2,NPY2R,PRNP,SLC16A1,ABCC8,TGFB2,TLR2,SYT7,C2CD5,SERGEF,ARFIP1,BCAS3,FBXW7,FERMT1,TAMALIN |
| 10 | GO Biological Processes | GO:0016049 | cell growth | -4.70 | 19 | ACVR1B,ACVRL1,ADRA1A,BCL6,CTNNB1,DPYSL2,PTK2B,SLIT1,TGFB2,KIAA0319,SLC23A2,ST7L,BIN3,PAK5,SMURF1,CDC73,JADE1,PLXNA4,ZFYVE27 |
| 11 | GO Biological Processes | GO:0042552 | myelination | -4.39 | 9 | CTSC,CLU,NF1,SCN8A,TG,TLR2,TSC1,NDRG1,MARVELD1 |
| 12 | KEGG Pathway | ko00730 | Thiamine metabolism | -4.32 | 4 | AK4,ALPL,NTPCR,AK8 |
| 13 | GO Biological Processes | GO:0007215 | glutamate receptor signaling pathway | -4.17 | 6 | DAGLA,PTK2B,GRM4,GRM5,PRNP,PLCB1 |
| 14 | GO Biological Processes | GO:0008285 | negative regulation of cell population proliferation | -4.15 | 24 | ACVRL1,ADRA1A,BCL6,BMP2,CTNNB1,PTK2B,GPLD1,NF1,PRNP,TBX5,TGFB2,TGFBR3,TLR2,TNFAIP3,TSC1,SCGB1A1,NDRG1,GLMN,TMEM131L,INTU,FBXW7,FERMT1,CDC73,BRIP1 |
| 15 | GO Biological Processes | GO:0002062 | chondrocyte differentiation | -4.14 | 8 | ACVRL1,BMP2,EXT1,GPLD1,LOXL2,WNT2B,CCN4,SLC39A14 |
| 16 | Canonical Pathways | M286 | PID TGFBR PATHWAY | -4.12 | 6 | CTNNB1,DAXX,PPP2R2A,TGFB2,TGFBR3,SMURF1 |
| 17 | GO Biological Processes | GO:0044851 | hair cycle phase | -4.12 | 3 | TGFB2,SPINK5,FERMT1 |
| 18 | GO Biological Processes | GO:0048514 | blood vessel morphogenesis | -3.96 | 22 | ACVRL1,CTNNB1,EGR3,PTK2B,GPLD1,NR4A1,HSPG2,JAK1,LEPR,LOXL2,NF1,PIK3C2A,TBX5,TGFB2,TGFBR3,TNFAIP3,VAV3,SPINK5,GLMN,BCAS3,FBXW7,JCAD |
| 19 | GO Biological Processes | GO:1900004 | negative regulation of serine-type endopeptidase activity | -3.92 | 3 | SPINK1,SPINK5,SPINK6 |
| 20 | GO Biological Processes | GO:0061098 | positive regulation of protein tyrosine kinase activity | -3.86 | 6 | ADRA1A,ADRA2A,GRM5,PRNP,FBXW7,IL34 |

Note:"Log10(P)" is the p-value in log base 10. "Count" is the number of genes in the user-provided lists with membership in the given ontology term.
